# Memory T-Waves: An Uncharted Territory in T-Wave Inversions

**DOI:** 10.7759/cureus.47620

**Published:** 2023-10-25

**Authors:** Munzir Malik, Muhammad S Farooqi, Rabail Karim, Saleem Khan, Irfan Ali Rind

**Affiliations:** 1 Acute Medicine, Wrexham Maelor Hospital, Wrexham, GBR; 2 Internal Medicine, Insight Hospital and Medical Center, Chicago, USA; 3 Neurology, The Walton Centre, Liverpool, GBR; 4 Medicine, Betsi Cadwaladr University Health Board, Wrexham, GBR; 5 Cardiology, Betsi Cadwaladr University Health Board, Wrexham, GBR

**Keywords:** cardiac chest pain, electrocardiography (ecg), ischemic heart disease, myocardial infarction, memory t waves

## Abstract

T-wave inversions on electrocardiograms (ECGs) can present a diagnostic challenge due to their association with various underlying causes. One less-explored cause is memory T-waves, a phenomenon characterized by T-wave inversions, often seen in chest and inferior leads, following a period of abnormal ventricular conduction. In this case report, we discuss the intriguing case of an 80-year-old woman who recently underwent percutaneous coronary intervention (PCI) for a myocardial infarction and subsequently developed memory T-waves. We are also discussing how important it can be to understand and recognize memory T-waves, as it will avoid further unnecessary tests and longer hospital stays.

## Introduction

Memory T-waves represent a unique ECG pattern that emerges once normal ventricular conduction is restored after a period of abnormal conduction [1]. This underreported phenomenon is critical to recognize, especially in patients with a history of cardiac conditions, to avoid unnecessary investigations and treatments. As Shvilkin et al. (2015) aptly put it, cardiac memory is indeed a "diagnostic tool in the making." Memory T-wave is a unique adaptive characteristic of the heart due to the changes of repolarization to the new activation pattern and manifesting by T-wave changes. Although this phenomenon is an adaptive reaction to the change in the ventricular activation sequence, it is often confused with acute coronary syndrome, which may require testing as an inpatient and prolongs hospital stay.

## Case presentation

An 80-year-old retired woman, who had previously enjoyed good health, presented to the emergency department (ED) two months after undergoing PCI for a myocardial infarction (MI) involving the left anterior descending artery (LAD). Her chief complaints were a general sense of unwellness and mild, non-radiating chest pain rated at 3/10 on the chest pain scale. The pain was different in character when compared with the pain of MI a couple of months ago. It also did not respond to glyceryl trinitrate (GTN) spray Initial blood tests, including a comprehensive metabolic panel (CMP), thyroid function tests (TFTs), and serial troponin measurements, returned unremarkable results. The patient's chest pain resolved with an analgesic (paracetamol).

**Figure 1:**
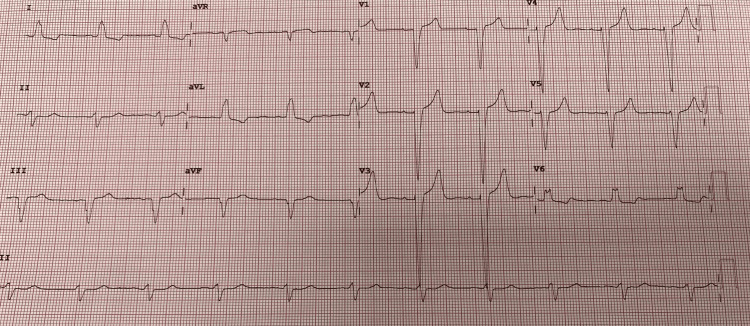
ECG at presentation showing a new left bundle branch block (LBBB)

**Figure 2:**
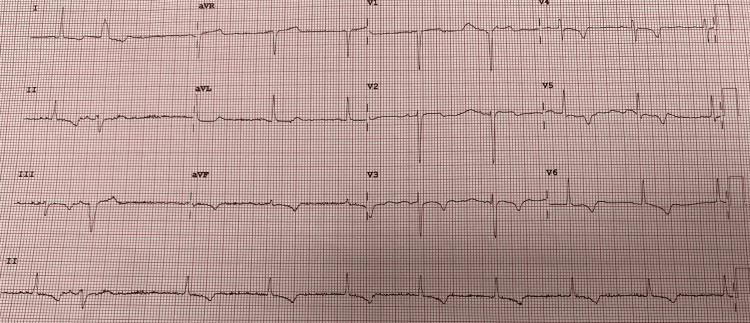
Subsequent ECG in next few hours showing memory T-waves

Upon admission, her ECG displayed intermittent left bundle branch block (LBBB) (Figure 1). The bedside echocardiogram did not reveal any regional wall motion abnormalities. The following day, she developed T-wave inversions in anterior and inferior leads while T-waves in leads I and AVL remained positive (Figure 2). These findings met the ECG criteria for memory T-waves, R Gunaseelan et al. showed that repeated troponin measurements remained within normal limits [2], and the patient remained asymptomatic.

Given the patient's previous myocardial infarction and her current medication regimen (dual antiplatelets, statins, and angiotensin-converting enzyme (ACE) inhibitor), she received reassurance and was discharged after ruling out other potential causes of T-wave inversions.

## Discussion

Memory T-waves is a term used to describe a phenomenon in electrocardiography (ECG) where the T-wave pattern on an ECG reflects the previous cardiac cycle’s events rather than the current cycle. This phenomenon is important to understand to avoid misdiagnosis and unnecessary investigations [2].

T-wave inversions on ECGs can be attributed to multiple factors, including electrolyte imbalances, medication side effects, pulmonary embolism, ventricular tachycardias, and hypothyroidism. However, in the acute setting, ischemia remains a primary concern [3]. One of the most underreported causes of T-wave inversions is memory T-waves, typically arising from transient conduction delays in the ventricles [4].

The ECG criteria for diagnosing memory T waves include T-wave inversions in precordial and inferior leads with positive or isoelectric T waves in leads I and AVL. These criteria have demonstrated a sensitivity of 100% and specificity of 96% when distinguishing memory T-waves from acute coronary syndrome (ACS) [5].

Identifying memory T-waves can be facilitated by recognizing ECGs with wide QRS complexes before the appearance of T-wave inversions in the same leads [4].

Memory T-waves represent an underreported and often overlooked phenomenon in electrocardiography (ECG). While they have been recognized and studied to some extent, they remain less explored than more common ECG abnormalities like ST-segment changes.

Memory T-waves pose a unique diagnostic challenge. Their resemblance to other ECG abnormalities, such as T-wave inversions due to ischemia or other cardiac disorders, can lead to confusion and misinterpretation. Recognizing memory T-waves is critical to avoid unnecessary investigations and treatments [6]. Identifying memory T-waves on an ECG can provide valuable clinical information about the patient's cardiac status and rhythm abnormalities. It may suggest underlying heart conditions or medication effects that need further evaluation and management [7]. One unique aspect of memory T-waves is their persistence even after the underlying condition is treated or the rhythm is normalized. This feature can help differentiate them from other ECG abnormalities and guide appropriate clinical management [8].

Despite their clinical relevance, memory T-waves remain an area of limited research. More comprehensive studies are needed to establish diagnostic criteria, prevalence rates, and long-term clinical outcomes associated with memory T-waves.

The presentation of memory T-waves can vary among individuals and can be influenced by factors such as underlying cardiac conditions, medication use, and electrolyte imbalances. This variability makes it challenging to establish universal diagnostic guidelines [1].

Mimicry of other ECG abnormalities

Memory T-waves can mimic other ECG abnormalities, such as Wolff-Parkinson-White syndrome (WPW). This similarity underscores the importance of careful interpretation and the need for additional diagnostic tests to confirm the presence of memory T-waves [4].

The treatment of memory T-waves involves addressing the underlying cause, which can be complex and multifactorial. This may require adjustments in medications, correction of electrolyte imbalances, or optimization of pacemaker settings, depending on the individual patient's circumstances.

In a normal cardiac cycle, the ECG waveform consists of the P-wave (atrial depolarization), QRS complex (ventricular depolarization), and T-wave (ventricular repolarization [9]. The T-wave typically follows the QRS complex and represents the recovery or repolarization phase of the ventricles. Memory T-waves are a deviation from the expected T-wave pattern seen in a standard ECG.

This phenomenon occurs when there is a delay in the repolarization process of the ventricles, causing the T-wave in one cardiac cycle to reflect the electrical changes from the previous cycle [1].

Important causes of memory T-waves

Bradyarrhythmias

Slow heart rhythms, such as bradycardia or heart block, can lead to memory T-waves. In these cases, the prolonged duration of the cardiac cycle allows the T-wave to be influenced by the previous cycle’s events [10].

Pacemaker Dysfunction

Patients with malfunctioning pacemakers or pacemaker-induced rhythms can also exhibit memory T-waves [4].

Certain Medications

Drugs that affect cardiac repolarization, such as antiarrhythmics, may contribute to this phenomenon.

Electrolyte Imbalances

Abnormal potassium, calcium, or magnesium levels can disrupt the normal repolarization process and lead to memory T-waves.

Identifying memory T-waves on an ECG can provide valuable information about the patient’s cardiac status and rhythm abnormalities [7]. It may suggest underlying heart conditions or medication effects that need further evaluation and management. Memory T-waves can sometimes mimic other ECG abnormalities, for example, WPW, so careful interpretation is essential [4]. The treatment of memory T-waves involves addressing the underlying cause. For example, correcting electrolyte imbalances, adjusting medications, or optimizing pacemaker settings may be necessary. In some cases, the resolution of memory T-waves can be a sign of improved cardiac function or rhythm stability [8].

## Conclusions

Memory T-waves remain a poorly understood aspect of cardiology, often leading to unnecessary investigations and treatments. Enhanced awareness of this phenomenon can help reduce unwarranted hospital admissions and alleviate patient anxiety. While distinguishing memory T-waves from T-wave inversions due to ischemia can be challenging, any doubts should be managed with a cautious approach, treating T-wave inversions as potentially ischemic until ruled out. Greater recognition of memory T waves can enhance patient care, reduce healthcare costs, and prevent undue patient distress.
